# Immune Regulatory Cells in Inflammation, Infection, Tumor, Metabolism, and Other Diseases 2019

**DOI:** 10.1155/2019/3182198

**Published:** 2019-10-23

**Authors:** Qingdong Guan, Cheng Xiao, Minggang Zhang

**Affiliations:** ^1^Cellular Therapy Laboratory, Manitoba Blood and Marrow Transplant Program, CancerCare Manitoba, Winnipeg, Canada; ^2^Department of Internal Medicine, University of Manitoba, Winnipeg, Canada; ^3^Department of Immunology, University of Manitoba, Winnipeg, Canada; ^4^Research Institute in Oncology and Hematology, CancerCare Manitoba, Winnipeg, Canada; ^5^Institute of Clinical Medicine, China-Japan Friendship Hospital, Beijing, China; ^6^Immunology Program, Memorial Sloan-Kettering Cancer Center, New York, USA

The immune regulatory components of the immune system, such as immune regulatory cells and regulatory cytokines, either natural or induced, play important roles in controlling a variety of physiological and pathological immune responses [1]. In the past years, great advances have been made in studying the physiopathological and therapeutic roles of immune regulator cells in inflammation, infection, tumor, transplantation, autoimmune diseases, and other diseases, such as mesenchymal stromal/stem cells (MSC) and regulatory T cells. MSC are being tested widely as a cellular therapy for autoimmune diseases and other diseases through modulating immune responses and/or promoting tissue repair [2–4]. Through proteomic analysis, we developed a reliable, rapid, and relevant potency assay for clinical grade MSC which is correlated with immunological function of MSC, and this may enhance the standardization of MSC cell products and ultimately promote the development of new MSC treatments [5, 6].

Myeloid-derived suppressor cells (MDSCs) are a heterogeneous population of immature cells that are imbalanced during cancer, transplantation, allergy, and other diseases and have a remarkable ability to suppress various T cell responses and to promote regulatory T cell expansion through multiple mechanisms [7–10]. The adoptive transfer of MDSCs sorted from animal models or generated in vitro could improve transplantation and autoimmune diseases, while targeting MDSC can improve cancer treatment. In this special issue, H. Ma and C.-Q. Xia summarized the phenotype and functional characteristics of MDSC and reviewed recent advances on their roles in the pathogenesis of autoimmune diseases and potential therapeutic applications.

Dendritic cells and macrophages are professional antigen-presenting cells with the ability to suppress or instigate immune responses and to bridge innate and adaptive immunity. In this issue, M. Mraz et al. evaluated the presence of HLA-DR^+^lineage^−^ DC and their subtype in peripheral blood and subcutaneous and epicardial adipose tissue in subjects with T2DM and with T2DM undergoing elective cardiac surgery and showed that T2DM decreased the amount of total DC but increased plasmacytoid DC in subcutaneous adipose tissue. H. Xu et al. showed that IL-10 deficiency restored the type 1 immune response through DC activation, thus providing better protection against TB infection. Using marrow cells from male FVB/N (control) and transgenic hypertensive animals, cells treated with M-CSF and subsequently with LPS and IFN-*γ* polarized into M1 macrophages and with IL-4 and IL-13 treatment cells polarized to M2 phenotype. P. A. M. Cavalcante et al. compared stimulated macrophages in vitro and found that cells from hypertensive mice were predisposed toward polarization to an M2 phenotype.

Proinflammatory cytokines and regulatory cytokines also play important roles in the physiopathology of diseases. In this special issue, E. Grudzinska et al. evaluated the levels of chemokines and growth factors produced by lymphocytes in the incompetent great saphenous vein. J. P. T. Guimaraes et al. explored the leukotriene involvement in the insulin receptor pathway and macrophage profiles in muscles from type I diabetic mice and showed that diabetic 5LO^−/−^ mice (lack of leukotriene synthesis) had a higher expression of insulin receptor and AKT phosphorylation and increased the expression of anti-inflammatory molecules IL-10, Arg1, and Ym1 and reduced the expression of proinflammatory cytokine IL-6 in muscle macrophage.

In response to IL-17, airway epithelial cells can produce antimicrobial proteins and neutrophil chemoattractants such as CXCR2 ligands. In order to understand how IL-17 exerts downstream effects on its target cells through epigenetic mechanisms, J. Luo et al. showed that IL-17-induced CXCR2 ligand productions are dependent on histone acetylation specifically through repressing HDAC5, and the recognition of acetylated histones plays a pivotal role. However, IL-17 responses were regulated differently by the DNA methylation mechanisms in specific lineages. By focusing on IFN signaling, H. M. Johnson et al. showed that ligand, IFN receptor, the JAKs, and the STATs all undergo endocytosis and ATP-dependent nuclear translocation to promoters of genes specifically activated by IFNs, which indicated that the vacuolar ATPase (V-ATPase) proton pump probably plays a key role in endosomal membrane crossing by IFNs for receptor cytoplasmic binding.

Innate lymphoid cells (ILCs) are a novel family of innate immune cells, which are shown to be critical for integrated mucosal immunity. L. Han et al. summarized studies about ILCs in inflammatory bowel disease (IBD), crosstalk of ILCs with intestinal microbiota, and the relationship of ILCs in enteric nervous system (ENS).

Using a guinea pig model of lipopolysaccharide- (LPS-) induced sudden sensorineural hearing loss (SSHL), L. Xia et al. studied the role of MAP kinase phosphatase-1 (MKP-1) and rosiglitazone (RSG) in glucocorticoid resistance/sensitivity. Severe hearing loss was observed in the LPS group, as opposed to the protection from hearing loss in the treatment of LPS, DEX, and RSG. A positive correlation was found between MKP-1 levels and protection from hearing loss. RSG and DEX synergistically influenced inner ear inflammation may result from impaired MKP-1 function in inner ear tissues, suggesting a novel target to develop potential therapeutics for inflammatory diseases of the inner ear.

Overall, we believe that these articles may improve our knowledge of immune regulatory cell-mediated immune mechanisms in infection, inflammation, tumor, and other diseases, providing insights into designing effective immunodiagnostic and potential therapeutic strategies.
